# Behavioral Domains and Physiological Characteristics in Young Adults: Findings from Integrated Seven-Day Self-Monitoring and Physiological Assessment

**DOI:** 10.3390/life16091520

**Published:** 2026-09-12

**Authors:** Stanislav Dadelo, Svetlana Danilenko

**Affiliations:** 1 Department of Entertainment Industries, Faculty of Creative Industries, Vilnius Gediminas Technical University, Saulėtekio al. 11, LT-10223 Vilnius, Lithuania; 2 Department of Mathematical Statistics, Faculty of Fundamental Sciences, Vilnius Gediminas Technical University, Saulėtekio al. 11, LT-10223 Vilnius, Lithuania; svetlana.danilenko@vilniustech.lt

**Keywords:** nutrition assessment, physical activity, sleep, body composition, physiological fitness, health monitoring, health promotion, university students

## Abstract

Objectives: This study examined patterns among six selected variables from seven-day self-monitored behavioral records and physiological characteristics and their cross-domain associations in young adults. Methods: The study included 157 university students aged 20–23 years (106 men, 51 women). Six behavioral variables derived from seven-day self-monitoring and seven physiological indicators were examined using sex-specific PCA, with component retention determined by parallel analysis. Cross-domain associations between the retained physiological and behavioral components were tested using Pearson correlations with Benjamini–Hochberg FDR correction. Results: Parallel analysis retained two physiological and two behavioral components in each sex. The physiological components represented body composition and cardiorespiratory fitness, while the behavioral components primarily reflected energy intake–expenditure and carbohydrate–protein patterns. Two of the eight cross-domain Pearson associations had raw *p*-values below 0.05, but neither remained statistically significant after FDR correction. Conclusions: The seven-day behavioral patterns and physiological characteristics showed limited correspondence. Seven-day self-monitoring can complement physiological assessment by documenting recent behavior, but should not be used as a substitute for direct assessment of physiological characteristics.

## 1. Introduction

Emerging adulthood is a critical period for establishing dietary habits, physical activity routines, sleep behaviors, and long-term health trajectories [1]. Lifestyle behaviors adopted during this developmental stage often persist into adulthood and may be associated with future physiological functioning and the risk of chronic disease. Nutrition, physical activity, sleep, and body composition jointly contribute to health-related outcomes and quality of life, and these behaviors may occur within broader patterns associated with physiological characteristics [2,3,4,5,6,7,8].

Contemporary health research increasingly considers lifestyle behaviors jointly rather than as isolated exposures [3,5,6,7,8]. Existing work has often focused on specific behavioral or physiological domains, including energy regulation [2] and longitudinal eating behavior [9]. Examining these sources together can show how recent behavioral patterns relate to body composition and physiological fitness without treating them as components of a single health construct [10].

Seven-day self-monitoring captures variation across weekdays and weekends that is not represented by a single-day assessment. Multi-day dietary records can account for day-to-day variation in intake, and seven-day food records have shown reproducibility for estimating average dietary intake [11,12]; comparable recording periods have also been used to assess physical activity under free-living conditions [13]. In the present study, nutrition, physical activity, sleep, and related behaviors were recorded concurrently in a participant-completed daily diary. Although self-reported records are subject to reporting and compliance limitations, they provide information on recent routines with little reliance on specialized equipment. In an educational setting, this form of self-observation is also relevant to health literacy [14], particularly given the prevalence of insufficient physical activity among young people [15].

This study aimed to characterize exploratory patterns among six selected variables from seven-day behavioral records and physiological indicators and to examine associations between the resulting component scores. The seven-day diary collected a broader set of measures of nutrition, physical activity, sleep, and related daily behaviors. The behavioral PCA was limited to energy intake, energy expenditure, carbohydrate intake, protein intake, vigorous physical activity, and food and water intake; the other recorded behavioral variables were retained for descriptive characterization. The physiological PCA comprised BMI, body fat percentage, muscle percentage, waist-to-hip ratio, resting heart rate, estimated V̇O_2_max, and Ruffier Index. Analyses were conducted separately in men and women to describe within-group covariance structures. Components occupying the same numerical position were not assumed to be equivalent across groups, and differences between the solutions were not interpreted as evidence of a sex effect.

## 2. Materials and Methods

### 2.1. Study Design and Setting

This cross-sectional study followed the STROBE guidelines, and all sex-disaggregated analyses followed the SAGER recommendations. The study was conducted at Vilnius Gediminas Technical University (VILNIUS TECH) in Lithuania under naturalistic, non-laboratory conditions. Participants completed a structured online self-monitoring diary on each of seven consecutive days while maintaining their usual daily routines. Seven days were selected to ensure each participant contributed observations from both weekdays and weekends, capturing within-week variation and reducing reliance on a single-day record [11,12,13]. The same predefined fields were completed each day. Participants recorded sleep duration; number and duration of meals; food and water intake; physical activity duration and intensity; and moderate- and vigorous-intensity activity sessions. Energy intake and macronutrient distribution were calculated from self-reported dietary intake, whereas daily energy expenditure was estimated from self-reported activity data. The variables and recording units are specified in Supplementary Table S1. Entries were reviewed daily for completeness and plausibility. During this review, missing values, implausible dietary entries, unrealistic self-recorded sleep durations, and inconsistencies between self-recorded activity and estimated energy expenditure were identified. Participants were contacted when substantial inconsistencies or incomplete records required clarification. After completion of the monitoring period, daily records were summarized across the seven days for the behavioral indicators used in the analyses. The diary was not externally validated against accelerometry, wearable devices, or controlled dietary assessment methods. Objective monitoring devices were not included because the protocol focused on participant-recorded information on nutrition, sleep, and physical activity during usual daily routines. Energy intake was calculated from self-recorded dietary intake, and energy expenditure was estimated from self-recorded activity rather than measured directly. This approach enabled the concurrent recording of several aspects of everyday behavior over a defined period, with limited disruption to participants. Data collection and processing adhered to Regulation (EU) 2016/679 [16].

### 2.2. Participants

A total of 157 university students, aged 20–23 years, participated (106 men and 51 women). Men and women had similar mean ages (21.36 ± 1.11 vs. 21.33 ± 1.09 years). Participants were recruited from health and physical education classes across the university. Inclusion criteria were ages between 20 and 23 years, participant-reported absence of known health conditions that would affect study participation, and no use of medications that affect metabolic or cardiovascular function. Health status was not assessed using a standardized clinical instrument. Exclusion criteria included pregnancy, recent musculoskeletal injuries, metabolic or cardiovascular conditions, and incomplete data submission.

Of the 180 students invited, 157 completed the process (response rate: 87.2%). Raw means and pooled standard deviations were used to calculate Cohen’s d, a descriptive standardized measure of the difference between men and women.

### 2.3. Procedures

Over seven days, participants maintained a structured online log of their daily routines. Functional fitness and anthropometric assessment were conducted using procedures consistent with established exercise-testing guidance and bioelectrical impedance methodology [17,18]. Logs were completed daily and monitored for consistency. Written informed consent was obtained from all participants before data collection. Physiological assessment was conducted during the same seven-day period as the behavioral self-monitoring. The original records do not specify the timing of these assessments relative to individual diary days.

### 2.4. Measures

Physiological Indicators: Physiological assessment included body mass index (BMI), waist-to-hip ratio (WHR), body fat percentage, muscle percentage, Ruffier Index, resting heart rate, and estimated V̇O_2_max [6,19,20,21]. Functional fitness and anthropometric assessments followed the methodological guidance cited above [17,18]. BMI was calculated as body weight (kg) divided by height squared (m^2^), and categorized according to WHO standard thresholds. Body fat and muscle percentages were obtained using bioelectrical impedance analysis according to the methodological principles described by Kyle et al. [18,22]. WHR was calculated as waist circumference divided by hip circumference following WHO guidance [23]. The device manufacturer, model, and pre-measurement conditions are not available in the study documentation. Resting heart rate and the Ruffier Index were obtained during the functional assessment, in accordance with the study protocol and established exercise-testing guidance [17]. V̇O_2_max was estimated rather than directly measured. The original methodology refers to both non-exercise and heart-rate-based estimation approaches [19,21], but the participant-level documentation does not specify the equation used to compute individual values. These missing specifications prevent exact reproduction of the BIA and V̇O_2_max procedures.

Behavioral Indicators (7-Day Averages): Behavioral data were derived from a structured diary completed on seven consecutive days. Participants recorded sleep duration (min/day), number of meals per day, average meal duration (min), daily food and water intake (g/day), physical activity duration and intensity, and moderate- and vigorous-intensity activity sessions. Daily energy intake (kcal/day) was calculated from self-recorded dietary intake using national food composition tables and cross-checked against Innih et al. [24]. Macronutrient intake was expressed as the percentage of total energy derived from carbohydrates, protein, and fat. Detailed instructions for identifying individual foods and estimating portion sizes are not available in the study documentation. Self-recorded physical activity was classified as light (<3 MET), moderate (3–6 MET), or vigorous (>6 MET) [25,26], with each category expressed as a proportion of recorded activity time. These data were not validated against accelerometer-based field assessment [27]. Daily energy expenditure (kcal/day) was estimated from self-recorded activity using a standard MET-based approach [25]; however, the participant-level computational steps needed to reproduce individual estimates are unavailable. Supplementary Table S1 provides the documented variables and recording units. Daily indicators were averaged across the seven days, whereas moderate- and vigorous-intensity activity sessions were summarized as weekly counts [11,12,13,28]. Thus, the behavioral dataset combined participant-recorded measures with values calculated or estimated from those records; no behavioral variable was measured with a device.

Variables Entered into Behavioral PCA: The exploratory behavioral PCA used six variables from the seven-day dataset: energy intake, energy expenditure, carbohydrate intake, protein intake, vigorous physical activity (>6 MET), and food and water intake. Sleep duration, number and duration of meals, dietary fat, light and moderate physical activity, and moderate- and vigorous-intensity activity sessions were used only for descriptive characterization. The analytical records identify the six PCA variables but do not document the original selection criteria. Their documented specification was therefore retained without applying a retrospective selection rule. The participant-to-variable ratio was approximately 17.7:1 in men (106 participants/6 variables) and 8.5:1 in women (51 participants/6 variables).

### 2.5. Data Preparation

Before analysis, the participant-level dataset was checked for completeness and consistency. Complete observations for all 21 variables were available for 157 participants (106 men and 51 women), with no missing values. During the seven-day monitoring period, participants were contacted when missing data, implausible values, or other inconsistencies required clarification. Only participants who completed the monitoring period and provided sufficiently complete behavioral records were retained. The original documentation states that values outside the ±3 SD range were excluded, but the exclusion log for the 180 invited students is unavailable. Consequently, the number of cases removed under this criterion, the variables involved, and the stage at which exclusion occurred cannot be reconstructed. Re-screening of the final dataset identified values exceeding ±3 SD from the sex-specific mean in 13 men and 5 women across one or more variables. These observations were retained because they were part of the final analytical sample, and no surviving information established that they had met the exclusion criterion when it was originally applied. No winsorization, imputation, or additional case exclusion was performed; the final analytical sample, therefore, remained 157 participants. Seven-day averages were calculated for the behavioral indicators before multivariate analysis. Principal component analysis and rotation followed established methodological guidance [29,30], and distributional normality was evaluated using the Shapiro–Wilk test [31].

### 2.6. Statistical Analysis

Principal Component Analyses: PCA was performed separately for men and women using the seven physiological indicators and six behavioral variables specified in Section 2.4. Sampling adequacy was evaluated with the Kaiser–Meyer–Olkin (KMO) measure and Bartlett’s test of sphericity. PCA was based on the correlation matrix and used Varimax rotation with Kaiser normalization [29,30]. Component retention was determined by parallel analysis using 20,000 normally distributed random datasets matched to the corresponding sample size and variable set [32]. Observed eigenvalues were compared with the 95th-percentile random eigenvalues; mean random eigenvalues are also reported in Supplementary Table S5. The 95th-percentile criterion retained two physiological and two behavioral components in both sex-specific analyses. The mean random-eigenvalue criterion supported a third behavioral component, whereas the 95th-percentile criterion did not. Primary two-component loading matrices are reported in Supplementary Tables S2 and S3, and the three-component solutions from the original extraction specification are presented as legacy sensitivity analyses in Supplementary Table S4. KMO values were 0.535 and 0.507 for the behavioral analyses and 0.606 and 0.589 for the physiological analyses in men and women, respectively. Bartlett’s test was significant in all four analyses (physiological: men, χ^2^(21) = 347.801, *p* < 0.001; women, χ^2^(21) = 178.075, *p* < 0.001; behavioral: men, χ^2^(15) = 168.212, *p* < 0.001; women, χ^2^(15) = 102.272, *p* < 0.001). Varimax rotation was iterated to convergence. Component scores were calculated from standardized variables using the regression scoring method and the corresponding rotated two-component solutions and were used in the cross-domain analyses.

As a supplementary analysis, physiological and behavioral variables were standardized separately within men and women before combining the groups (*N* = 157). PCA was then applied to the within-sex-standardized values using the same seven physiological indicators and six behavioral variables. This removed between-group mean differences while retaining within-group variation. Extraction and Varimax rotation followed the primary analysis, and component retention was determined by parallel analysis with 20,000 random datasets and the 95th-percentile random-eigenvalue criterion [32]. Complete results are reported in Supplementary Table S8.

Additional Analytical Procedures: Distributional normality was assessed separately in men and women using the Shapiro–Wilk test [31]. Variables deviating from normality in either group were compared using the Mann–Whitney U test [33]; Welch’s independent-samples *t*-test was used when neither group deviated from normality. Benjamini–Hochberg false discovery rate correction was applied as a single family across the 21 between-group comparisons in Table 1, with the complete multiplicity calculation reported in Supplementary Table S10. Cohen’s d, based on group means and pooled standard deviations, was reported as a descriptive standardized effect-size measure [34]. All tests were two-tailed.

Software: PCA, KMO, and Bartlett tests, communalities, and Mann–Whitney U tests were conducted in IBM SPSS Statistics, version 26.0 (IBM Corp., Armonk, NY, USA) [35]. The available SPSS output and participant-level data were sufficient to verify the correlation-matrix PCA specification, eigenvalues, sampling-adequacy statistics, rotated component matrices, input matrices, and extraction results. Because the original component-score specification is unavailable, regression-based scores were recalculated for the two retained component solutions and used in subsequent cross-domain analyses.

Additional Association Analyses: Cross-domain relationships were examined separately in men and women using regression-based scores for the two physiological and two behavioral components retained in the primary analyses. Because behavioral component composition and ordering differed between the sex-specific solutions, numerical component positions were not treated as equivalent constructs across groups. Pearson correlations were calculated for the four physiological–behavioral combinations within each group, yielding eight primary two-tailed tests. Benjamini–Hochberg FDR correction was applied across these eight correlations. The same associations were examined with Spearman rank correlations as a sensitivity analysis, with a separate FDR correction across the eight tests. Pearson results are reported in Supplementary Table S6 and Spearman results in Supplementary Table S7. Statistical significance after correction was defined as FDR-adjusted *p* < 0.05.

Ethics Statement: The study was conducted in 2024 as part of the officially approved course “Sports, Health and Lifestyle” (course code KIPIB93006) at Vilnius Gediminas Technical University. During the educational process, students completed project-based activities involving health self-monitoring, dietary assessment, physical activity assessment, lifestyle evaluation, and related self-assessment tasks. Participation in the research using the collected data was voluntary. The data were anonymized before analysis, and no personally identifiable information was collected, stored, or processed. All participants provided written informed consent before their data were included in the study. No medical treatment, clinical intervention, biological sampling, or experimental manipulation was performed. On 15 June 2026, the Faculty of Creative Industries, Vilnius Gediminas Technical University, issued a subsequent institutional confirmation concerning the status of the educational activity conducted in 2024. The confirmation states that under the University’s approved study-program regulations and course-approval procedures, separate approval from an Institutional Review Board or Research Ethics Committee was not required for this type of educational activity. This confirmation was issued retrospectively and did not constitute prior ethical approval of the 2024 activity. The study procedures were conducted in accordance with the principles of the Declaration of Helsinki and Regulation (EU) 2016/679 [16].

## 3. Results

### 3.1. Descriptive Statistics

Table 1 presents descriptive statistics for the 21 physiological, dietary, and activity-related variables available in the participant-level dataset, separately for men and women. Between-group comparisons were performed using the Mann–Whitney U test when either group deviated from normality and Welch’s independent-samples *t*-test otherwise. Two-tailed raw *p*-values and Benjamini–Hochberg FDR-adjusted *p*-values are reported together with Cohen’s d. These univariate comparisons describe differences in the observed variables within the present sample and were not used to infer sex differences in the multivariate component structures or cross-domain associations. After Benjamini–Hochberg FDR adjustment across the 21 comparisons, differences remained statistically significant for BMI, WHR, body fat percentage, body muscle percentage, Ruffier Index, resting heart rate, V̇O_2_max, food and water intake, energy intake, and energy expenditure. Carbohydrate intake differed at the unadjusted level (raw *p* = 0.03467571) but not after FDR correction (adjusted *p* = 0.06619908). No FDR-adjusted differences were detected for the remaining behavioral variables.

### 3.2. Primary Physiological PCA: Two-Component Solutions

Parallel analysis using the 95th-percentile random-eigenvalue criterion supported two physiological components in both men and women (Supplementary Table S5); the corresponding primary loading matrices are reproduced in Supplementary Table S2. The retained components explained 66.229% of the total variance in men and 68.995% in women. In both groups, Component 1 was defined primarily by BMI, body fat percentage, and muscle percentage, which together represent body composition. In contrast, Component 2 was defined primarily by resting heart rate, the Ruffier Index, and estimated V̇O_2_max, which represent cardiorespiratory fitness. WHR loaded moderately on Component 1 in men and showed weaker loadings across the two components in women. The third observed eigenvalue did not exceed the corresponding 95th-percentile random eigenvalue in either group. The sex-specific loading matrices are presented in Table 2.

### 3.3. Primary Behavioral PCA: Two-Component Exploratory Solutions

Parallel analysis using the 95th-percentile random-eigenvalue criterion supported two components among the six selected behavioral variables in both men and women (Supplementary Table S5); the corresponding primary loading matrices are reproduced in Supplementary Table S3. The retained components explained 62.067% of the total variance in men and 61.692% in women. In men, Component 1 was characterized primarily by energy intake, energy expenditure, and food and water intake, whereas Component 2 reflected higher carbohydrate and lower protein intake. In women, Component 1 reflected higher carbohydrate and lower protein intake, whereas Component 2 was characterized primarily by energy intake and food and water intake. Vigorous physical activity did not show a dominant loading on either of the retained components. The third observed eigenvalue was 1.074 in men and 1.064 in women, below the corresponding 95th-percentile random eigenvalues of 1.117 and 1.163. The sex-specific loading matrices are presented in Table 3.

### 3.4. Cross-Domain Associations: Eight Primary Tests

Cross-domain associations were examined using regression-based component scores from the two-component physiological and behavioral solutions retained by parallel analysis. Four combinations of physiological–behavioral components were examined separately in men and women, yielding eight primary Pearson tests. These results are presented in Table 4. Two associations had raw *p*-values below 0.05: Phys1 × Beh1 in men (*r* = 0.2355, raw *p* = 0.01511) and Phys1 × Beh1 in women (*r* = −0.3131, raw *p* = 0.02528). Neither remained statistically significant after Benjamini–Hochberg FDR correction (FDR-adjusted *p* = 0.10113 for both).

### 3.5. Sensitivity Analyses

Spearman rank correlations were calculated for the same eight cross-domain associations examined in the primary Pearson analysis. One association had a raw *p*-value below 0.05, Phys1 × Beh1 in men (ρ = 0.2062, raw *p* = 0.03395). Still, none met the significance threshold after a separate Benjamini–Hochberg FDR correction across the eight Spearman tests (Supplementary Table S7). The corresponding association in women was weaker under Spearman’s rank correlation (ρ = −0.2530, raw *p* = 0.07321).

The original three-component physiological and behavioral PCA solutions were retained as legacy sensitivity analyses (Supplementary Table S4). The three-component physiological solutions explained 79.649% of the total variance in men and 82.613% in women, but parallel analysis did not support retaining the third physiological component. The three-component behavioral solutions explained 79.975% in men and 79.422% in women; their third components were supported by the mean random-eigenvalue criterion but not by the 95th-percentile criterion used for the primary analyses. Cross-domain correlations based on these legacy three-component solutions are reported in Supplementary Table S9 and were not used for primary inference.

A further sensitivity analysis standardized each physiological and behavioral variable separately within men and women before combining the standardized values (*N* = 157). Parallel analysis using the 95th-percentile criterion retained three physiological components and two behavioral components. The combined within-sex-standardized physiological solution separated body composition, cardiorespiratory indicators, and a WHR-related component, whereas the behavioral solution retained the energy intake–expenditure and carbohydrate–protein patterns (Supplementary Table S8). These results were treated as sensitivity analyses and did not replace the primary sex-specific two-component solutions.

## 4. Discussion

The principal finding was the limited correspondence between the behavioral patterns derived from the seven-day records and the physiological characteristics assessed in this sample [8,36,37,38,39,40,41].

### 4.1. Physiological and Behavioral Component Structures

Parallel analysis supported two physiological components in both sex-specific analyses, representing body composition and cardiorespiratory fitness. The broadly similar loading patterns in men and women are consistent with evidence that health and well-being in young adulthood involve interrelated dimensions of body composition, physical fitness, dietary practices, and exercise-related adaptation [42,43,44,45,46]. Alternative solutions showed that WHR was the least stable element of the physiological structure, emerging as a separate component under some analytical specifications.

The exploratory PCA of the six selected behavioral variables identified patterns primarily reflecting energy intake–expenditure and carbohydrate–protein intake, although their ordering differed between men and women. The weaker stability of an additional behavioral component, together with modest KMO values and the smaller female subgroup, supports cautious interpretation of the behavioral structure. Differences between male and female structures remain descriptive because structural equivalence was not formally tested, and the analyses do not distinguish sex-related biological influences from broader sex- and gender-related variation [47,48].

### 4.2. Cross-Domain Associations Between Behavioral and Physiological Components

Because the behavioral components differed in composition and ordering between the sex-specific solutions, their numerical positions were not treated as equivalent across groups.

The different temporal scales of the measurements may partly explain the observed cross-domain associations. The behavioral components summarized six selected variables recorded over seven days, whereas body composition and cardiorespiratory fitness reflect longer-term physiological processes. Short-term records of intake and activity may therefore correspond only weakly with characteristics shaped by accumulated exposure, adaptation, and individual variation. Although energy balance is central to long-term body-weight regulation, its relationship with body composition is also influenced by adaptive expenditure, energy compensation, metabolic requirements, body size, and behavioral history [36,37,38].

At the unadjusted level, body composition correlated positively with the energy intake–expenditure pattern in men (*r* = 0.2355, raw *p* = 0.01511) and negatively with the carbohydrate–protein pattern in women (*r* = −0.3131, raw *p* = 0.02528). Both associations had an FDR-adjusted *p*-value of 0.10113 and therefore did not meet the corrected significance threshold. Because association strengths and component structures were not formally compared between groups, these within-group findings do not demonstrate differences between men and women.

### 4.3. Theoretical Implications

Health-related behaviors occur in heterogeneous combinations and vary with individual, environmental, and social conditions [8,40,41,49]. Systems-based approaches likewise recognize that related health dimensions need not follow a uniform pattern [50].

### 4.4. Practical Implications

**Figure 1:**
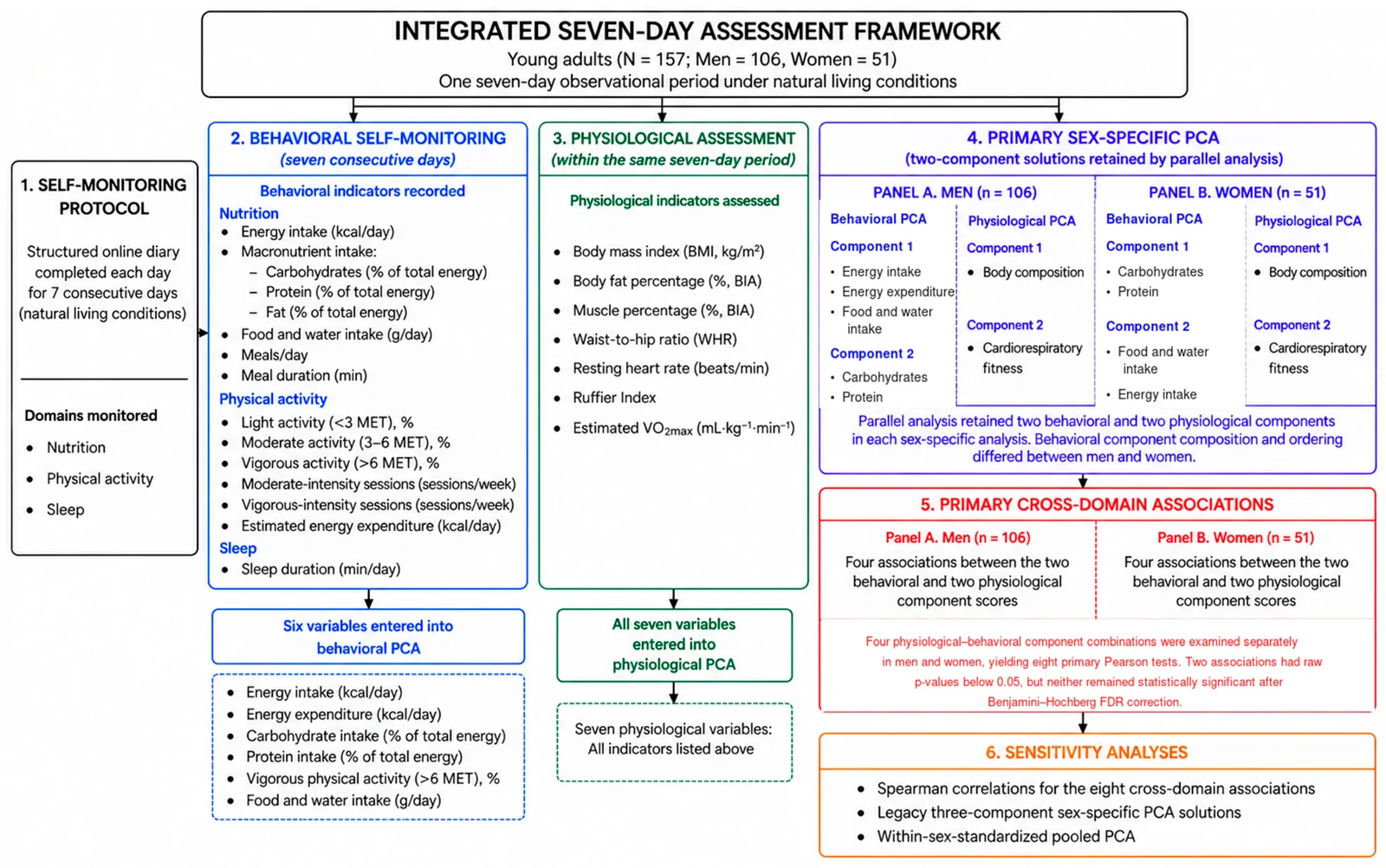
Seven-day behavioral self-monitoring, physiological assessment, and analytical framework among young adults. Note. The seven-day diary collected nutrition, physical activity, sleep, and related daily measures. Six selected behavioral variables and seven physiological indicators were included in sex-specific PCAs. Parallel analysis retained two behavioral and two physiological components in each group for the primary cross-domain analysis. Four combinations of components were examined separately in men and women, yielding eight Pearson tests. Additional PCA solutions and Spearman correlations were examined as sensitivity analyses. Blue denotes behavioral self-monitoring and behavioral PCA, green denotes physiological assessment and physiological PCA, red denotes the primary cross-domain associations, and orange denotes sensitivity analyses; black denotes the overall protocol.

The seven-day diary provided information on nutrition, physical activity, sleep, and related behaviors under habitual conditions without requiring continuous specialized monitoring (Figure 1). Its minimal equipment requirements make it feasible for educational and other real-world settings, where recording everyday behavior can support reflection on health-related choices. Periodic assessment of body composition and physiological fitness provides complementary information that is not available from the diary alone [51]. The weak cross-domain associations observed here indicate that participant-recorded behavior and variables calculated or estimated from those records should not be used to infer physiological status. Cardiorespiratory fitness [52] and sleep, dietary, and body-composition characteristics [53] should therefore be assessed independently when these outcomes are of interest. When greater measurement precision is required, objective activity assessment and established dietary methods should be preferred [27,28,54].

### 4.5. Limitations

Several limitations should be considered when interpreting the findings. The behavioral data were based on participant self-monitoring and are therefore subject to reporting error. Dietary intake may be under- or overreported, while self-recorded physical activity and sleep duration may be inaccurate [54]. Daily review allowed incomplete or implausible entries to be clarified, but could not eliminate errors in the underlying records. The lack of objective validation also affects values calculated from dietary records or estimated from reported activity.

Incomplete methodological documentation limits the reproducibility of some measurements and analytical procedures and introduces uncertainty regarding their exact implementation [18,19,21,24,25]. Although the preserved participant-level dataset supports reanalysis, results that depend on these procedures should be interpreted with this constraint in mind.

The PCA findings should also be interpreted as exploratory. Sampling adequacy for the behavioral analyses was modest (KMO = 0.535 in men and 0.507 in women), and estimates for women were based on the smaller subgroup. Component retention was determined by parallel analysis using the 95th-percentile random-eigenvalue criterion [32], with alternative solutions examined in sensitivity analyses. The identified structures, therefore, describe patterns in this sample rather than established latent constructs.

Interpretation of the cross-domain findings is further constrained by the cross-sectional design and the different time scales of the measurements. The seven-day records characterize recent behavior, whereas body composition and cardiorespiratory fitness reflect processes developing over longer periods. The short behavioral observation window may consequently attenuate associations between these domains, and the present data do not establish temporal relationships or directionality.

The sample comprised 20–23-year-old students recruited from health and physical education classes at a single university. This recruitment setting may have favored students with a greater interest in physical activity or health-related behaviors, thereby contributing to compliance with the seven-day protocol. Selection bias cannot therefore be excluded, and the findings should not be assumed to represent the broader young-adult or university-student population.

## 5. Conclusions

Seven-day behavioral records and physiological assessment captured largely distinct aspects of health in this sample, with the retained behavioral and physiological component structures showing limited correspondence. Cross-domain associations were modest, and none remained statistically significant after FDR correction. These findings indicate that behavioral patterns recorded over seven days should not be used as proxies for physiological status, which reflects characteristics developing over different time scales. Seven-day self-monitoring can therefore complement physiological assessment by documenting recent behavior, but it cannot replace direct physiological assessment.

## Figures and Tables

**Table 1:** Descriptive statistics by sex, raw and Benjamini–Hochberg FDR-adjusted *p*-values, and effect sizes.

Indicator	Unit	Men (*n* = 106)	Women (*n* = 51)	Raw *p*	BH-FDR *p*	Cohen’s d
BMI	kg/m^2^	23.68 ± 2.52	21.42 ± 3.21	0.00000204	0.00000785	0.82
WHR	units	0.94 ± 0.06	0.79 ± 0.07	3.65 × 10^−19^	3.83 × 10^−18^	2.23
Body fat percentage	%	16.46 ± 3.53	20.97 ± 6.30	0.00000224	0.00000785	−0.98
Body muscle percentage	%	48.18 ± 2.73	43.32 ± 3.35	1.74 × 10^−14^	1.21 × 10^−13^	1.65
Ruffier Index	units	5.70 ± 2.70	7.30 ± 2.85	0.00117906	0.00275113	−0.58
Resting heart rate	bpm	66.55 ± 8.56	70.51 ± 8.00	0.00541070	0.01136248	−0.47
V̇O_2_max	mL/kg/min	47.32 ± 6.92	42.98 ± 5.63	0.00013296	0.00039633	0.66
Sleep duration	min/day	479.80 ± 47.00	488.55 ± 53.55	0.07855227	0.11782841	−0.18
Meals per day	units	3.81 ± 0.56	3.92 ± 0.82	0.35412896	0.44773826	−0.17
Meal duration	min	13.88 ± 4.12	15.08 ± 6.27	0.77480296	0.85636117	−0.24
Food and water intake	g/day	1697.76 ± 352.50	1484.21 ± 486.85	0.00015098	0.00039633	0.53
Energy intake	kcal/day	2501.88 ± 389.33	1793.34 ± 237.93	1.29 × 10^−20^	2.72 × 10^−19^	2.04
Carbohydrate intake	% TE	57.53 ± 7.04	60.07 ± 6.94	0.03467571	0.06619908	−0.36
Protein intake	% TE	23.38 ± 5.28	21.91 ± 4.88	0.19791465	0.27708051	0.29
Fat intake (% of total energy)	% TE	19.10 ± 5.38	18.02 ± 4.18	0.05763336	0.10085838	0.21
Energy expenditure	kcal/day	2725.97 ± 393.14	2275.45 ± 269.49	5.91 × 10^−12^	3.10 × 10^−11^	1.26
Light activity (MET < 3)	%	74.48 ± 13.22	77.13 ± 11.34	0.50585120	0.59015973	−0.21
Moderate activity (MET 3–6)	%	15.96 ± 11.89	15.21 ± 9.87	0.98056181	0.98056181	0.07
Vigorous activity (MET > 6)	%	9.56 ± 8.96	7.66 ± 7.26	0.36245478	0.44773826	0.22
Moderate-activity sessions	sessions/week	5.42 ± 3.38	7.22 ± 5.03	0.06609741	0.10677273	−0.45
Vigorous-activity sessions	sessions/week	2.53 ± 2.47	2.33 ± 1.99	0.97421205	0.98056181	0.08

Note: Values are mean ± SD. Distributional normality was assessed separately in men and women using the Shapiro–Wilk test. Between-group comparisons were performed using the Mann–Whitney U test when either group deviated from normality and Welch’s independent-samples *t*-test otherwise. Raw *p*-values are two-tailed. Benjamini–Hochberg FDR adjustment was applied as a single family across all 21 comparisons. Cohen’s d was calculated using the pooled standard deviation; positive values indicate higher values in men and negative values indicate higher values in women.

**Table 2:** Sex-specific Varimax-rotated component loadings for the two physiological components retained in the primary analyses.

**Panel A. Men (*n* = 106)**		
**Indicator**	**Component 1**	**Component 2**
BMI	0.926	−0.042
WHR	0.449	−0.001
Body fat %	0.748	−0.070
Muscle %	−0.890	0.063
Ruffier Index	0.057	0.837
Resting heart rate	−0.064	0.919
V̇O_2_max	0.132	−0.803
**Panel B. Women (*n* = 51)**		
**Indicator**	**Component 1**	**Component 2**
BMI	0.955	−0.005
WHR	0.287	−0.337
Body fat %	0.876	0.050
Muscle %	−0.866	−0.091
Ruffier Index	0.080	0.768
Resting heart rate	0.083	0.893
V̇O_2_max	−0.034	−0.889

Note: Extraction method: principal component analysis. Rotation method: Varimax with Kaiser normalization, iterated to convergence. Two components were retained in each sex-specific analysis according to parallel analysis using the 95th-percentile random-eigenvalue criterion [32]. The retained components explained 66.229% of the total variance in men and 68.995% in women. Component 1 represented body composition, and Component 2 cardiorespiratory fitness. The three-component solutions obtained under the original extraction specification are reported in the Supplementary Materials as part of the sensitivity analyses.

**Table 3:** Sex-specific Varimax-rotated component loadings for the two behavioral components retained in the primary analyses.

**Panel A. Men (*n* = 106)**		
**Indicator**	**Component 1**	**Component 2**
Energy intake	0.875	−0.064
Energy expenditure	0.842	−0.079
Carbohydrates	0.124	0.889
Protein	0.026	−0.902
Vigorous PA (>6 MET)	0.285	−0.299
Food and water intake	0.654	0.145
**Panel B. Women (*n* = 51)**		
**Indicator**	**Component 1**	**Component 2**
Protein	−0.944	0.022
Carbohydrates	0.936	0.032
Food and water intake	0.080	0.914
Energy intake	0.290	0.873
Vigorous PA (>6 MET)	0.090	−0.261
Energy expenditure	−0.295	−0.284

Note: Extraction method: principal component analysis. Rotation method: Varimax with Kaiser normalization. Two components were retained according to parallel analysis using the 95th-percentile random-eigenvalue criterion [32]. The retained components explained 62.067% of the total variance in men and 61.692% in women. Sampling adequacy was modest (KMO = 0.535 for men and KMO = 0.507 for women). Bartlett’s tests of sphericity were significant in both groups (men: χ^2^(15) = 168.212, *p* < 0.001; women: χ^2^(15) = 102.272, *p* < 0.001). The three-component solutions obtained under the original extraction specification are reported in the Supplementary Materials as part of the sensitivity analyses.

**Table 4:** Cross-domain correlations between physiological and sex-specific behavioral components with raw and Benjamini–Hochberg FDR-adjusted *p*-values.

Physiological × Behavioral	r	Raw *p*	BH-FDR *p*
Men Phys1 × Beh1	0.2355	0.01511	0.10113
Men Phys1 × Beh2	0.0133	0.89227	0.89227
Men Phys2 × Beh1	−0.1403	0.15150	0.40399
Men Phys2 × Beh2	0.0642	0.51341	0.80375
Women Phys1 × Beh1	−0.3131	0.02528	0.10113
Women Phys1 × Beh2	−0.0746	0.60282	0.80375
Women Phys2 × Beh1	0.1316	0.35715	0.71430
Women Phys2 × Beh2	−0.0336	0.81510	0.89227

Note: Pearson correlation coefficients are reported with two-tailed raw *p*-values and Benjamini–Hochberg FDR-adjusted *p*-values computed across all eight cross-domain tests. Phys1 represents body composition, and Phys2 represents cardiorespiratory fitness, in both groups. In men, Beh1 represents the component characterized primarily by energy intake, energy expenditure, and food and water intake, whereas Beh2 represents the carbohydrate–protein pattern. In women, Beh1 represents the carbohydrate–protein pattern and Beh2 the component characterized primarily by energy intake and food and water intake. Components with the same numerical position are therefore not assumed to represent equivalent behavioral constructs across sex groups.

## Data Availability

The datasets generated and analyzed in this study are available from the corresponding author upon reasonable request.

## Supplementary Materials

The following supporting information can be downloaded at: https://www.mdpi.com/article/10.3390/life16091520/s1, Table S1: Structure, recording units, and derivation of seven-day self-monitoring variables; Table S2: Primary two-component sex-specific physiological PCA loading matrices; Table S3: Primary two-component sex-specific behavioral PCA loading matrices; Table S4: Legacy three-component sex-specific physiological and behavioral PCA loading matrices; Table S5: Observed eigenvalues, percentage of explained variance, and parallel-analysis retention criteria for the sex-specific physiological and behavioral PCA; Table S6: Pearson cross-domain associations with raw and Benjamini–Hochberg FDR-adjusted *p*-values for the eight primary tests; Table S7: Spearman sensitivity analysis of the eight cross-domain associations with raw and Benjamini–Hochberg FDR-adjusted *p*-values; Table S8: Physiological and behavioral PCA sensitivity analysis of the combined within-sex-standardized data (*N* = 157); Table S9: Legacy cross-domain Pearson correlations based on the original three-component physiological and behavioral PCA solutions; Table S10: Benjamini–Hochberg FDR adjustment for the 21 between-group comparisons reported in Table 1.

## Abbreviations

The following abbreviations are used in this manuscript:

ACSM	American College of Sports Medicine
BMI	Body Mass Index
BFP	Body Fat Percentage
KMO	Kaiser–Meyer–Olkin
MET	Metabolic Equivalent of Task
PA	Physical Activity
PCA	Principal Component Analysis
RHR	Resting Heart Rate
RI	Ruffier Index
V̇O_2_max	Maximal Oxygen Uptake
WHO	World Health Organization
WHR	Waist-to-Hip Ratio
