# Supplementary material for: Behavioral Domains and Physiological Characteristics in Young Adults: Findings from Integrated Seven-Day Self-Monitoring and Physiological Assessment
Source: Life (Basel). 2026 Sep 12;16(9):1520. doi: 10.3390/life16091520 (PMC13608643; doi:10.3390/life16091520)
Supplement: Supplementary file 1 [file life-16-01520-s001.zip › life-4539821-supplementary.pdf]

**Supplementary Materials**

The following supporting information accompanies the manuscript: Table S1: Structure, recording units, and derivation of seven-day self-monitoring variables; Table S2: Primary two-component sex-specific physiological PCA loading matrices; Table S3: Primary two-component sex-specific behavioral PCA loading matrices; Table S4: Legacy three-component sex-specific physiological and behavioral PCA loading matrices; Table S5: Observed eigenvalues, percentage of explained variance, and parallel-analysis retention criteria for the sex-specific physiological and behavioral PCA; Table S6: Pearson cross-domain associations with raw and Benjamini–Hochberg FDR-adjusted p-values for the eight primary tests; Table S7: Spearman sensitivity analysis of the eight cross-domain associations with raw and Benjamini–Hochberg FDR-adjusted p-values; Table S8: Physiological and behavioral PCA sensitivity analysis of the combined within-sex-standardized data; Table S9: Legacy cross-domain Pearson correlations based on the original three-component physiological and behavioral PCA solutions; Table S10: Benjamini–Hochberg FDR adjustment for the 21 between-group comparisons reported in Table 1.

**Table S1.** Structure, recording units, and derivation of seven-day self-monitoring variables.

| Diary field | Recording unit or format | Seven-day processing |
|---|---|---|
| Sleep duration | min/day | Arithmetic mean across the seven-day monitoring period |
| Number of meals | number/day | Arithmetic mean across the seven-day monitoring period |
| Meal duration | min | Arithmetic mean across the seven-day monitoring period |
| Food and water intake | g/day | Total recorded daily amount; arithmetic mean across the seven-day monitoring period |
| Energy intake | kcal/day | Calculated from recorded dietary intake using national food composition tables; arithmetic mean across the seven-day monitoring period |
| Carbohydrates | % of total energy | Percentage of daily energy intake derived from carbohydrates; arithmetic mean across the seven-day monitoring period |
| Protein | % of total energy | Percentage of daily energy intake derived from protein; arithmetic mean across the seven-day monitoring period |
| Fat | % of total energy | Percentage of daily energy intake derived from fat; arithmetic mean across the seven-day monitoring period |
| Light physical activity | % of recorded activity time (<3 MET) | Proportion of recorded activity time classified as light intensity across the seven-day monitoring period |
| Moderate physical activity | % of recorded activity time (3–6 MET) | Proportion of recorded activity time classified as moderate intensity across the seven-day monitoring period |
| Vigorous physical activity | % of recorded activity time (>6 MET) | Proportion of recorded activity time classified as vigorous intensity across the seven-day monitoring period |
| Moderate-intensity activity sessions | sessions/week | Total number of recorded moderate-intensity sessions during the seven-day monitoring period |
| Vigorous-intensity activity sessions | sessions/week | Total number of recorded vigorous-intensity sessions during the seven-day monitoring period |
| Estimated energy expenditure | kcal/day | Estimated from recorded activity duration and intensity using standard MET-based calculations; arithmetic mean across the seven-day monitoring period |

*Note: Participants completed the same structured diary on each of seven consecutive days while maintaining their usual routines. Daily records were reviewed for completeness and plausibility, and participants were contacted when substantial inconsistencies or incomplete entries required clarification. Energy intake was calculated from the recorded dietary intake using national food composition tables and cross-checked against Innih et al. [24]. Physical activity was recorded by duration and intensity, with intensity classified as light (<3 MET), moderate (3–6 MET), or vigorous (>6 MET) [25,26]. Energy expenditure was estimated from the recorded activity duration and intensity using standard MET-based calculations [25]. The archived study documentation does not provide sufficient detail to determine retrospectively how participants identified individual foods or estimated portion sizes, or to reconstruct the participant-level energy-expenditure calculation beyond its documented MET-based basis. These procedures are therefore not specified beyond the information preserved in the original study records.*

**Table S2.** Primary two-component sex-specific physiological PCA loading matrices.

**Panel A. Men (n = 106)**

| Indicator | Component 1 | Component 2 |
|---|---|---|
| BMI | .926 | −.042 |
| WHR | .449 | −.001 |
| Body fat % | .748 | −.070 |
| Muscle % | −.890 | .063 |
| Ruffier Index | .057 | .837 |
| Resting heart rate | −.064 | .919 |
| $VO_2$max | .132 | −.803 |

**Panel B. Women (n = 51)**

| Indicator | Component 1 | Component 2 |
|---|---|---|
| BMI | .955 | −.005 |
| WHR | .287 | −.337 |
| Body fat % | .876 | .050 |
| Muscle % | −.866 | −.091 |
| Ruffier Index | .080 | .768 |
| Resting heart rate | .083 | .893 |
| $VO_2$max | −.034 | −.889 |

Note: Extraction method: principal component analysis. Rotation method: Varimax with Kaiser normalization, iterated to convergence. Two components were retained in each sex-specific analysis according to parallel analysis using the 95th-percentile random-eigenvalue criterion [32]. The retained components explained 66.229% of total variance in men and 68.995% in women. Component 1 represents body composition and Component 2 cardiorespiratory fitness.

**Table S3.** Primary two-component sex-specific behavioral PCA loading matrices.

**Panel A. Men (n = 106)**

| **Indicator** | **Component 1** | **Component 2** |
|---|---|---|
| Energy intake | −.064 | .875 |
| Energy expenditure | −.079 | .842 |
| Carbohydrates | .889 | .124 |
| Protein | −.902 | .026 |
| Vigorous PA (>6 MET) | −.299 | .285 |
| Food and water intake | .145 | .654 |

**Panel B. Women (n = 51)**

| **Indicator** | **Component 1** | **Component 2** |
|---|---|---|
| Protein | .022 | −.944 |
| Carbohydrates | .032 | .936 |
| Food and water intake | .914 | .080 |
| Energy intake | .873 | .290 |
| Vigorous PA (>6 MET) | −.261 | .090 |
| Energy expenditure | −.284 | −.295 |

Note: *Extraction method: principal component analysis. Rotation method: Varimax with Kaiser normalization. Two components were retained according to parallel analysis using the 95th-percentile random-eigenvalue criterion [32]. The retained components explained 62.067% of total variance in men and 61.692% in women. Sampling adequacy was modest (KMO = 0.535 for men and KMO = 0.507 for women). Bartlett's tests of sphericity were significant in both groups (men:* $\chi^2(15) = 168.212$, $p < .001$; *women:* $\chi^2(15) = 102.272$, $p < .001$).

**Table S4.** Legacy three-component sex-specific physiological and behavioral PCA loading matrices.

**Panel A. Physiological PCA, men (n = 106)**

| Indicator | Component 1 | Component 2 | Component 3 |
|---|---|---|---|
| BMI | .961 | −.035 | .045 |
| Muscle % | −.934 | .056 | −.010 |
| Body fat % | .678 | −.069 | .333 |
| Resting heart rate | −.032 | .920 | −.118 |
| Ruffier Index | −.006 | .835 | .192 |
| $\dot{V}O_2max$ | .109 | −.804 | .099 |
| WHR | .158 | −.012 | .954 |

**Panel B. Physiological PCA, women (n = 51)**

| Indicator | Component 1 | Component 2 | Component 3 |
|---|---|---|---|
| BMI | .915 | .051 | .282 |
| Body fat % | .900 | .034 | −.022 |
| Muscle % | −.894 | −.070 | .045 |
| Resting heart rate | .048 | .921 | .010 |
| $\dot{V}O_2max$ | −.040 | −.871 | .179 |
| Ruffier Index | .055 | .785 | −.015 |
| WHR | .087 | −.112 | .981 |

*Note: Extraction method: principal component analysis. Rotation method: Varimax with Kaiser normalization. The three-component solutions explained 79.649% of total variance in men and 82.613% in women. Parallel analysis supported two physiological components in both groups under both the mean and 95th-percentile random-eigenvalue criteria. The third component was therefore not included in the primary sex-specific solutions, and the three-component matrices are presented here as legacy sensitivity analyses [32].*

**Table S4.** Legacy three-component sex-specific physiological and behavioral PCA loading matrices (continued).

**Panel C. Behavioral PCA, men (n = 106)**

| Indicator | Component 1 | Component 2 | Component 3 |
|---|---|---|---|
| Energy intake | .915 | −.016 | .111 |
| Energy expenditure | .915 | −.040 | .035 |
| Carbohydrates | .015 | .907 | .081 |
| Protein | .053 | −.891 | .132 |
| Vigorous PA (>6 MET) | −.063 | −.195 | .877 |
| Food and water intake | .400 | .244 | .660 |

**Panel D. Behavioral PCA, women (n = 51)**

| Indicator | Component 1 | Component 2 | Component 3 |
|---|---|---|---|
| Protein | −.940 | −.055 | .077 |
| Carbohydrates | .929 | .131 | −.011 |
| Food and water intake | −.007 | .946 | −.048 |
| Energy intake | .204 | .910 | −.099 |
| Vigorous PA (>6 MET) | .141 | −.004 | .836 |
| Energy expenditure | −.247 | −.126 | .650 |

*Note: Extraction method: principal component analysis. Rotation method: Varimax with Kaiser normalization. Six behavioral variables were included in each sex-specific analysis. The three-component solutions explained 79.975% of total variance in men and 79.422% in women. Parallel analysis supported three components in both groups under the mean random-eigenvalue criterion but two components under the 95th-percentile criterion [32]. Accordingly, the primary analyses retained two components, while the three-component matrices are presented here as legacy sensitivity analyses. Sampling adequacy was modest (KMO = 0.535 in men and KMO = 0.507 in women).*

**Table S5.** Observed eigenvalues, percentage of explained variance, and parallel-analysis retention criteria for the sex-specific physiological and behavioral PCA.

| **Analysis** | **Component** | **Observed eigenvalue** | **Explained variance (%)** | **Mean random eigenvalue** | **95th-percentile random eigenvalue** | **Retained, mean criterion** | **Retained, 95th-percentile criterion** |
|---|---|---|---|---|---|---|---|
| Physiological PCA, men (n = 106; 7 variables) | 1 | 2.607 | 37.249 | 1.381 | 1.525 | Yes | Yes |
| | 2 | 2.029 | 28.980 | 1.216 | 1.313 | Yes | Yes |
| | 3 | 0.939 | 13.421 | 1.093 | 1.169 | No | No |
| Physiological PCA, women (n = 51; 7 variables) | 1 | 2.633 | 37.618 | 1.564 | 1.781 | Yes | Yes |
| | 2 | 2.196 | 31.377 | 1.311 | 1.453 | Yes | Yes |
| | 3 | 0.953 | 13.618 | 1.127 | 1.239 | No | No |
| Behavioral PCA, men (n = 106; 6 variables) | 1 | 2.002 | 33.372 | 1.333 | 1.471 | Yes | Yes |
| | 2 | 1.722 | 28.695 | 1.167 | 1.261 | Yes | Yes |
| | 3 | 1.074 | 17.908 | 1.044 | 1.117 | Yes | No |
| Behavioral PCA, women (n = 51; 6 variables) | 1 | 2.260 | 37.665 | 1.489 | 1.700 | Yes | Yes |
| | 2 | 1.442 | 24.027 | 1.240 | 1.374 | Yes | Yes |
| | 3 | 1.064 | 17.730 | 1.057 | 1.163 | Yes | No |

*Note. Explained variance refers to the percentage of total variance associated with each observed initial eigenvalue before rotation. Parallel analysis was based on 20,000 normally distributed random datasets matched to the sample size and number of variables in each sex-specific analysis. Observed eigenvalues were compared with the mean and 95th-percentile random eigenvalues. Under the mean criterion, two physiological components and three behavioral components were retained in both men and women. Under the 95th-percentile criterion, two components were retained in all four analyses. Components were retained when the observed eigenvalue exceeded the corresponding random-data criterion [32].*

**Table S6.** Pearson cross-domain associations with raw and Benjamini–Hochberg FDR-adjusted p-values for the eight primary tests.

| Physiological × Behavioral | r | Raw p | BH-FDR p |
|---|---|---|---|
| Men Phys1 × Beh1 | .2355 | .01511 | .10113 |
| Men Phys1 × Beh2 | .0133 | .89227 | .89227 |
| Men Phys2 × Beh1 | −.1403 | .15150 | .40399 |
| Men Phys2 × Beh2 | .0642 | .51341 | .80375 |
| Women Phys1 × Beh1 | −.3131 | .02528 | .10113 |
| Women Phys1 × Beh2 | −.0746 | .60282 | .80375 |
| Women Phys2 × Beh1 | .1316 | .35715 | .71430 |
| Women Phys2 × Beh2 | −.0336 | .81510 | .89227 |

*Note: Pearson correlation coefficients are reported with two-tailed raw p-values and Benjamini–Hochberg FDR-adjusted p-values calculated across all eight primary cross-domain tests. No association remained statistically significant after FDR correction (adjusted p < .05). Because behavioral component composition and ordering differed between men and women, components with the same numerical position should not be interpreted as equivalent across sex groups.*

**Table S7.** Spearman sensitivity analysis of the eight cross-domain associations with raw and Benjamini–Hochberg FDR-adjusted p-values.

| **Physiological × Behavioral** | **Spearman ϱ** | **Raw p** | **BH-FDR p** |
|---|---|---|---|
| Men Phys1 × Beh1 | .2062 | .03395 | .27157 |
| Men Phys1 × Beh2 | −.0762 | .43747 | .58329 |
| Men Phys2 × Beh1 | −.1270 | .19443 | .51292 |
| Men Phys2 × Beh2 | .1112 | .25646 | .51292 |
| Women Phys1 × Beh1 | −.2530 | .07321 | .29286 |
| Women Phys1 × Beh2 | −.0642 | .65465 | .65465 |
| Women Phys2 × Beh1 | .1163 | .41642 | .58329 |
| Women Phys2 × Beh2 | −.0794 | .57984 | .65465 |

*Note. Spearman rank correlations were calculated as a sensitivity analysis for the same eight associations examined in the primary Pearson analysis. Benjamini–Hochberg FDR adjustment was applied separately across the eight Spearman tests. Men Phys1 × Beh1 reached raw $p < .05$, but no Spearman association remained statistically significant after FDR correction. The two Pearson associations with raw $p < .05$ were weaker under Spearman estimation: Men Phys1 × Beh1 changed from $r = .2355$ to $\rho = .2062$, and Women Phys1 × Beh1 from $r = -.3131$ to $\rho = -.2530$.*

**Table S8.** Physiological and behavioral PCA sensitivity analysis of the combined within-sex-standardized data (N = 157).

**Panel A. Physiological PCA**

| Indicator | Component 1 | Component 2 | Component 3 |
|---|---|---|---|
| BMI | .551 | −.087 | .758 |
| Body fat % | .878 | .060 | .037 |
| Muscle % | −.907 | −.095 | .116 |
| Resting heart rate | .022 | .922 | −.054 |
| Ruffier Index | .093 | .834 | −.014 |
| $VO_2max$ | −.046 | −.821 | .213 |
| WHR | −.307 | −.159 | .892 |

**Panel B. Behavioral PCA**

| Indicator | Component 1 | Component 2 |
|---|---|---|
| Energy intake | .905 | −.048 |
| Energy expenditure | .807 | −.160 |
| Carbohydrates | .008 | .915 |
| Protein | .076 | −.908 |
| Vigorous PA (>6 MET) | .281 | −.195 |
| Food and water intake | .689 | .187 |

*Note. Before pooling the sample, each variable was standardized separately within men and women. PCA was then performed on the combined within-sex standardized values. This procedure removed between-group mean differences from the pooled input variables while retaining within-group variation. Extraction was based on principal component analysis with Varimax rotation and Kaiser normalization. Component retention was evaluated by parallel analysis using 20,000 random datasets and the 95th-percentile random-eigenvalue criterion [32].*

**Table S9.** Legacy cross-domain Pearson correlations based on the original three-component physiological and behavioral PCA solutions.

| Physiological × Behavioral | r | Raw p | BH-FDR p |
|---|---|---|---|
| Men Phys1 × Beh1 | .2285 | .01849 | .30162 |
| Men Phys1 × Beh2 | .0672 | .49374 | .84354 |
| Men Phys1 × Beh3 | .0238 | .80828 | .88414 |
| Men Phys2 × Beh1 | −.1725 | .07696 | .34634 |
| Men Phys2 × Beh2 | .0639 | .51550 | .84354 |
| Men Phys2 × Beh3 | .0285 | .77188 | .88414 |
| Men Phys3 × Beh1 | .0789 | .42133 | .84354 |
| Men Phys3 × Beh2 | −.1186 | .22597 | .81350 |
| Men Phys3 × Beh3 | .0376 | .70183 | .88414 |
| Women Phys1 × Beh1 | −.2983 | .03351 | .30162 |
| Women Phys1 × Beh2 | −.0008 | .99556 | .99556 |
| Women Phys1 × Beh3 | .1545 | .27888 | .83664 |
| Women Phys2 × Beh1 | .1260 | .37812 | .84354 |
| Women Phys2 × Beh2 | −.0994 | .48781 | .84354 |
| Women Phys2 × Beh3 | −.0299 | .83503 | .88414 |
| Women Phys3 × Beh1 | −.0614 | .66886 | .88414 |
| Women Phys3 × Beh2 | −.2712 | .05427 | .32565 |
| Women Phys3 × Beh3 | .0576 | .68829 | .88414 |

*Note: The original cross-domain analysis comprised nine physiological–behavioral component combinations within each sex group, yielding 18 Pearson correlations. These results are retained as a legacy sensitivity analysis to document the associations obtained from the original three-component extraction specification. They are not part of the primary cross-domain analysis, which was based on the two physiological and two behavioral components retained by parallel analysis using the 95th-percentile random-eigenvalue criterion. Pearson correlation coefficients are reported with two-tailed raw p-values and Benjamini–Hochberg FDR-adjusted p-values computed across all 18 cross-domain tests. Because the composition and ordering of behavioral components differed between men and women, components at the same numerical position are not equivalent across sex groups.*

**Table S10.** Benjamini–Hochberg FDR adjustment for the 21 between-group comparisons reported in Table 1.

| Indicator | Raw p | BH-FDR p |
|---|---|---|
| BMI | .00000204 | .00000785 |
| WHR | $3.65 \times 10^{-19}$ | $3.83 \times 10^{-18}$ |
| Body fat percentage | .00000224 | .00000785 |
| Body muscle percentage | $1.74 \times 10^{-14}$ | $1.21 \times 10^{-13}$ |
| Ruffier Index | .00117906 | .00275113 |
| Resting heart rate | .00541070 | .01136248 |
| $VO_2$max | .00013296 | .00039633 |
| Sleep duration | .07855227 | .11782841 |
| Meals per day | .35412896 | .44773826 |
| Meal duration | .77480296 | .85636117 |
| Food and water intake | .00015098 | .00039633 |
| Energy intake | $1.29 \times 10^{-20}$ | $2.72 \times 10^{-19}$ |
| Carbohydrate intake | .03467571 | .06619908 |
| Protein intake | .19791465 | .27708051 |
| Fat intake | .05763336 | .10085838 |
| Energy expenditure | $5.91 \times 10^{-12}$ | $3.10 \times 10^{-11}$ |
| Light activity (MET < 3) | .50585120 | .59015973 |
| Moderate activity (MET 3–6) | .98056181 | .98056181 |
| Vigorous activity (MET > 6) | .36245478 | .44773826 |
| Moderate-activity sessions | .06609741 | .10677273 |
| Vigorous-activity sessions | .97421205 | .98056181 |

*Note: Benjamini–Hochberg false discovery rate adjustment was applied as a single family across all 21 between-group comparisons reported in Table 1. Raw p-values are two-tailed. FDR-adjusted p-values below .05 were considered statistically significant.*
